# Safety and Efficacy of Pressurized Intra-Thoracic Aerosol Chemotherapy in Non-Small Cell Lung Cancer Pleural Carcinomatosis: Preliminary Results of a Pilot Study

**DOI:** 10.3390/mps8030051

**Published:** 2025-05-14

**Authors:** Maria Giovanna Mastromarino, Vittorio Aprile, Gianmarco Elia, Diana Bacchin, Alessandra Lenzini, Stylianos Korasidis, Marcello Carlo Ambrogi, Silvia Martina Ferrari, Poupak Fallahi, Marco Lucchi

**Affiliations:** 1 Division of Thoracic Surgery, Cardiac, Thoracic and Vascular Department, University Hospital of Pisa, Via Paradisa 2, 56124 Pisa, Italy; mgmastromarino@gmail.com (M.G.M.);; 2 Department of Surgical, Medical and Molecular Pathology and Critical Care Medicine, University of Pisa, Via Roma, 56124 Pisa, Italy; 3 Department of Translational Research and New Technologies in Medicine and Surgery, University of Pisa, Via Roma, 56124 Pisa, Italy

**Keywords:** non-small cell lung cancer, pleural carcinomatosis, intrathoracic chemotherapy, pleurodesis, malignant pleural effusion, primary cell cultures

## Abstract

Pleural carcinomatosis (PC) and malignant pleural effusion (MPE) affect up to 20% of patients with non-small cell lung cancer (NSCLC) and are usually synonymous with poor prognosis. Pressurized Intra-Thoracic Aerosol Chemotherapy (PITAC) is a novel and promising technique to control MPE in PC-NSCLC. This pilot study aimed to assess the feasibility, safety, and efficacy of PITAC in terms of palliative pleurodesis and evaluate the local antineoplastic control by analyzing patient-derived primary cell cultures. From January to December 2023, seven patients underwent PITAC with tailored doses of cisplatin and doxorubicin. There were four males and three females, with a median age of 65 (IQR:19) years. No operating room contamination by aerosolized chemotherapeutics was observed. No intraoperative complications occurred, and 30-day mortality was nil. One patient developed a postoperative prolonged air leak. The median chest tube stay was 2 (IQR:2) days, and the median hospital stay was 4 (IQR:2) days. No systemic toxicity nor hypersensitivity to chemotherapeutics were observed. All patients developed effective pleurodesis in 30 days. Cell cultures obtained from biopsy of PC-NSCLC sampled before PITAC formed confluent and monolayer sheets of attached tumor cells, while after 30 min from PITAC, cultures exhibited a significant reduction in the cancer cells’ growth. Effective pleurodesis was observed three and six months after surgery in all patients. PITAC is a safe and effective technique to control MPE recurrence and might revolutionize loco-regional therapy for PC-NSCLC. Further research should assess its oncological role.

## 1. Introduction

Non-small cell lung cancer (NSCLC) is the leading cause of cancer-related mortality worldwide [1]. Due to its tendency to metastasize, pleural carcinomatosis (PC) and malignant pleural effusion (MPE) are present in up to 20% of NSCLC cases [2]. Systemic therapy remains the cornerstone of treatment in advanced stages. However, despite significant advancements in targeted therapy and immunotherapy, most patients with metastatic disease have a poor prognosis [3]. Immunotherapy may help slow disease progression; however, its impact on survival is limited in patients with MPE that is not effectively controlled through appropriate interventions [2]. In such cases, treatment is primarily palliative, aiming to alleviate clinical symptoms [4]. Pleurodesis, which induces adhesion between the pleural layers, is used to prevent MPE recurrence. To date, the most commonly used sclerosing agent is sterile surgical talc (hydrated magnesium silicate) [5]. In recent years, intrapleural perfusion with various chemotherapeutic agents has been explored in an effort to combine antineoplastic effects with traditional pleurodesis [6,7,8]. However, oncological outcomes remain inconsistent, and a standardized approach has yet to be established.

The use of Hyperthermic Intrathoracic Chemotherapy (HITHOC) is limited in the palliative management of MPE; instead, it is utilized as part of surgery-based multimodal therapy with curative intent to improve local tumor control in primary or secondary malignancies involving serous surfaces [6,8,9].

In this scenario, Pressurized Intra-Thoracic Aerosol Chemotherapy (PITAC) might represent a novel therapeutic approach [10]. This technique was inherited from abdominal surgery, where it is carried out to treat unresectable peritoneal metastasis (PtM) with promising results in local disease control [11,12]. Theoretically, PITAC could provide both pleurodesis and an antineoplastic effect by delivering a high-pressure aerosolized chemotherapy into the thoracic cavity [13]. Most data on the efficacy of antineoplastic drugs on PC have been obtained through preclinical research on primary cancer cell cultures. Similarly, several studies have shown that in vitro chemosensitivity correlates with clinical responses, suggesting that these cultures could support the development of personalized treatments for patients with advanced-stage NSCLC [14,15].

We designed a phase II, non-randomized, open-label pilot study with the following objectives:

(a) to assess the feasibility, safety, and preliminary efficacy of PITAC, a novel surgical technique that combines the advantages of surgery and intrathoracic chemotherapy, in inducing pleurodesis and reducing MPE recurrence as part of a multimodal, personalized treatment strategy for PC-NSCLC patients;
(b) to evaluate the oncological efficacy of aerosolized chemotherapeutic agents in terms of local disease control, by analyzing patient-derived primary cancer cell cultures obtained from PC-NSCLC biopsies before and after PITAC.

## 2. Materials and Methods

### 2.1. Patients and Study Endpoint

All patients affected by a confirmed or highly suspected (according to the pre-operative imaging) PC-NSCLC with MPE, requiring a Video-Assisted Thoracoscopic Surgery (VATS) operation with diagnostic and staging intent, relieve symptoms, and induce pleurodesis, were enrolled.

Eligibility criteria included the following: age between 18 and 80 years; good performance status (Eastern Oncology Cooperative Group (ECOG) score ≤ 2) suitable for general anesthesia [16]; survival expectancy > 3 months; liver, renal and cardiopulmonary function parameters within 10% of the normal range; diagnosis of PC-NSCLC (in suspected cases, pathologic confirmation has been obtained by intraoperative frozen section). All enrolled patients were preoperatively discussed in the multidisciplinary tumor board (MTB).

Exclusion criteria were as follows: stage IVB (multiple extrathoracic metastases in one or more organs), according to the eight edition of TNM classification for lung cancer [17], and intraoperative findings of trapped lung.

PITAC efficacy was evaluated according to the World Health Organization(WHO)’s criteria for the Treatment Response of MPE after surgery [18]:

- Complete Response (CR): no pleural effusion and symptom-free for at least four weeks;
- Partial Response (PR): less than 50% effusion recurrence, asymptomatic, no thoracentesis needed within four weeks;
- Stable Disease (SD): more than 50% effusion recurrence or unchanged effusion, symptomatic, thoracentesis needed within four weeks;
- Progression Disease (PD): more than 25% increase in pleural effusion.

Objective treatment response of MPE after PITAC included CR and PR and was assessed in an outpatient setting 30 days, 3 months, and 6 months after surgery, with chest X-ray and/or computed tomography (CT) scan, planned according to the follow-up (FUP) protocol.

Intra and perioperative morbidity, according to the Common Terminology Criteria for Adverse Events (CTCAE v5.0) [19], and intraoperative and 30-day mortality were recorded and reported.

The Institutional Review Board has approved the study. All methods conformed to the principles of the Declaration of Helsinki. Each patient provided written informed consent for collecting, analyzing, and publishing this study’s prospectively obtained anonymized data.

This manuscript was written according to the Consolidated Standards of Reporting Trials (CONSORT) Statement [20]. The CONSORT checklist is available as the Supplemental File S1.

The next step of this ongoing phase 2 trial will be the assessment of the oncological efficacy of PITAC at the locoregional level, specifically in terms of local (intrapleural) disease progression, according to the RECIST (Response Evaluation Criteria in Solid Tumors) version 1.1. This will be evaluated using chest CT scans scheduled 8–12 weeks post-surgery, in accordance with routine FUP conducted by a dedicated oncologist, as per international guidelines [3,21].

### 2.2. Medication

The selection of chemotherapy agents was made according to the available literature on a similar procedure for PtM. Dedicated pharmacologists prepared cytostatic solutions on medical prescription according to the Body Surface Area (BSA), calculated with the Boyd formula [22]. The drug regimen consisted of Cisplatin (Iketon, Milan, Italy) at doses of 10.5 mg/m^2^ in 150 mL NaCl 0.9% and Doxorubicin (Pharmacia, Milan, Italy) at doses of 2.1 mg/m^2^ in 50 mL NaCl 0.9%.

### 2.3. Technique

**Figure 1:**
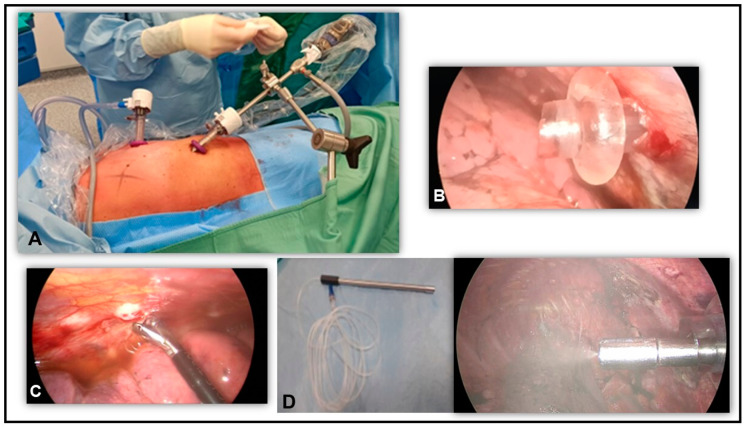
(**A**) Patient in lateral decubitus position, with two 12 mm balloon trocars, one in the VII ICS in the mid-axillary line and the second one in the V ICS in the anterior axillary line. (**B**) Intraoperative view of 12 mm balloon trocar. (**C**) Intraoperative picture of parietal pleural biopsy. (**D**) CE-certified nebulizer and intra-thoracic nebulization of chemotherapeutic agents.

**Figure 2:**
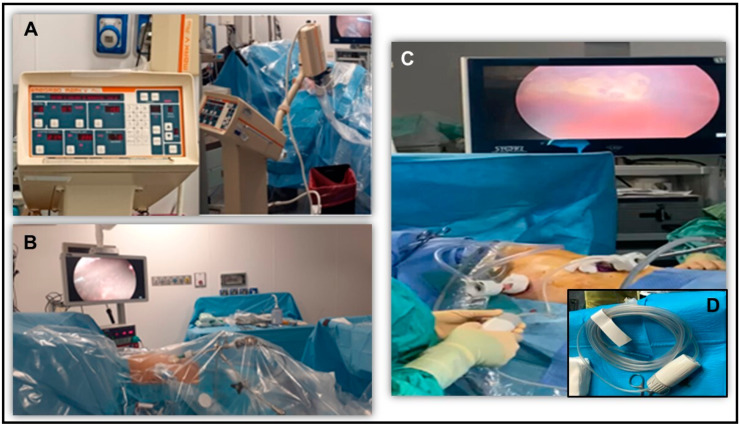
(**A**) High-pressure injector connected to CE-certified nebulizer to consecutively aerosolize cisplatin and doxorubicin into the pleural cavity at 0.7 mL/s flow with a maximal upstream pressure of 1.517 × 10^6^ Pa. (**B**) The nebulization procedure is remote-controlled from outside the operating room. (**C**) Intraoperative view of residual molecules of the toxic aerosol aspirated using a closed surgical smoke evacuation system. (**D**) Detail of surgical smoke evacuation system with two micro-particle filters.

The patient was positioned in lateral decubitus under general anesthesia with double-lumen intubation. After the insertion of two 12 mm balloon trocars (Applied Medical, Rancho Santa Margarita, CA, USA) into the chest wall (VII intercostal space (ICS) in the mid-axillary line and V ICS in the anterior axillary line, Figure 1A,B), a standard thoracoscopy was performed. Balloon trocars were needed to create a closed system and avoid dispersion of chemotherapy aerosol outside the thoracic cavity. After MPE aspiration and lysis of eventual pleural adhesions, multiple parietal pleural biopsies were sampled on macroscopic lesions or random pleural points (Figure 1C). A pleural sample was sent for frozen analysis if preoperative diagnosis was unavailable. Only confirmed cases of PC-NSCLC proceeded with PITAC. A dedicated checklist was reviewed to ensure all safety aspects were covered before chemotherapy administration. An intrathoracic pressure of 12 mmHg CO_2_ was established, and a CE-certified disposable nebulizer (RegerGmbH, Villingendorf, Germany) was inserted through a trocar and connected to a high-pressure injector (Figure 1D). The patient was covered with a sterile drape and all the staff left the operating room (OR) to avoid eventual chemotherapy exposure. Using a remote control, cisplatin and doxorubicin were consecutively aerosolized into the pleural cavity at 0.7 mL/s flow with a maximal upstream pressure of 1.517 × 10^6^ Pa (Figure 2A). Vital signs and nebulization procedure were remote-controlled, although acceding to the OR should be safe with no inhalation risk given laminar airflow (Figure 2B). The system maintained a steady state for 30 min (constant intrathoracic pressure of 12 mmHg CO_2_) to enhance drug penetration. After this period, the staff entered the OR wearing aerosol masks to remove residual toxic aerosol using a closed surgical smoke evacuation system with micro-particle filters (Figure 2C,D). Finally, the trocars were removed, and two chest drainages were placed as standard procedure.

### 2.4. Primary Cell Cultures

To evaluate the antineoplastic effect of the high-pressure aerosolized chemotherapy, PCs were sampled both before (time-0) and after drugs nebulization (time-30 min after PITAC); these latter pleural biopsies were obtained from the pleura surrounding the previous biopsy. Samples were submitted for primary cell cultures within 30 min from the collection. The areas of necrosis were removed, and solid tissue was minced and reduced to fragments of about 1 mm. In order to digest the collagen and release tumor cells the fragments were incubated in cell culture medium in the presence of collagenase type I at 200 U/mL concentration for one hour [23]. Since primary cell cultures derive from surgical biopsies, to avoid the possible interference in the conducted analysis given by the presence of macrophages, red blood cells and lymphocytes, which are, in general, the main contaminants, all the primary cells were used within 6–10 passages. Cells were cultured in RPMI with 2 mM glutamine, 100 U/mL penicillin/100 μg/mL streptomycin (Sigma-Aldrich, St. Louis, MO, USA), and 10% fetal bovine serum (FBS) and maintained at 37 °C and 5% CO_2_ [24,25].

In order to assess the reduction in cell growth, it was evaluated by cell number counting [26]. Cells were plated at a density of 13,000 cells/well in 24-well tissue culture plates in complete medium and counted using a hemocytometer.

### 2.5. Statistical Analysis

Data were collected and extracted from our prospective institutional database and medical records. Continuous variables were defined as mean (standard deviation, SD) or median (interquartile range, IQR) where appropriate. Categorical variables were expressed as absolute numbers unless stated otherwise. This paper aims to present the preliminary results of a pilot study. Then, given the small number of patients, no comparison analyses were conducted with a control group. Neither survival probability nor predictive factors were evaluated because statistical significance could not be obtained according to the underpowered sample size.

## 3. Results

From January to December 2023, forty patients affected by a confirmed or suspected PC with MPE underwent VATS at our tertiary care university center. Seven patients were enrolled in this study and underwent PITAC procedure following a confirmed diagnosis of PC-NSCLC. The remaining 33 patients had PC associated with other malignancies. All seven enrolled patients had not received any prior local treatments, such as surgery or radiation therapy, before undergoing PITAC. The cohort included four males and three females, with a median age of 65 (IQR:19) years. The pre-operative characteristics of the patients are summarized in Table 1.

Six patients had not received prior systemic chemotherapy, while one patient had undergone two cycles of immune checkpoint inhibitors (ICIs) before surgery. Four patients had a previously confirmed diagnosis of PC-NSCLC, whereas in three cases, pleural involvement was confirmed intraoperatively via frozen section analysis. The mean intra-operative pleural effusion volume was 1180.0 (SD: 785.5) mL. The mean operative time was 125.0 (SD: 27.2) minutes. No OR contamination by aerosolized chemotherapeutic drugs was observed.

**Figure 3:**
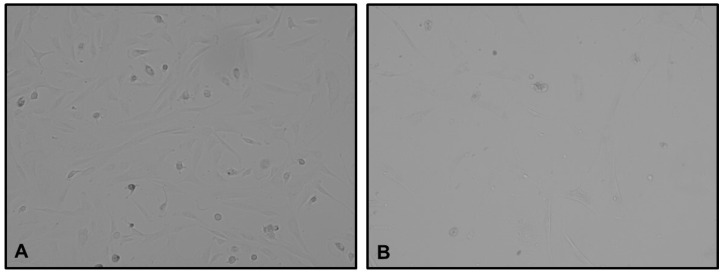
(**A**) Example of time 0 cell cultures formed confluent and mainly monolayer sheets of medium-sized attached tumor cells (magnification, 10×). (**B**) Example of time 30 min after PITAC cell cultures exhibited a significant reduction in cancer cell growth (magnification, 10×).

PC biopsies were obtained both before and after chemotherapy nebulization. Tumor tissues were used to establish patient-derived primary bi-dimensional (2D) cell cultures of PC-NSCLC. The time 0 cultures formed confluent, primarily monolayer sheets of medium-sized attached tumor cells (Figure 3A). In contrast, time 30 min after PITAC cultures showed a significant reduction in cancer cell growth (Figure 3B).

No major or minor intraoperative complications occurred, and there was no 30-day mortality. One patient developed a prolonged post-operative air leak, resulting from intraoperative adhesiolysis required to release a trapped lung; this was managed conservatively with chest tube drainage for 10 days. No postoperative complications were observed in the remaining six patients. The median chest tube length was 2 (IQR:2) days, and the median hospital stay was 4 (IQR:2) days.

No systemic toxicity or hypersensitivity to chemotherapeutic agents was observed during hospitalization or throughout early and mid-term FUP. Pathological examination confirmed pleural metastases from lung adenocarcinoma in all patients. Immunohistochemical and molecular characteristics are summarized in Table 2.

All patients developed effective pleurodesis within 30 days, as confirmed by serial post-operative chest X-rays. According to the WHO Criteria for the Treatment Response of MPE after surgery, five patients demonstrated a CR, and two showed a PR. The same responses were maintained at 3 and 6 months postoperatively, with effective pleurodesis observed in the entire cohort throughout the FUP period. All patients received postoperative first-line systemic therapy: four were treated with platinum-based chemotherapy combined with ICIs; two patients with EGFR mutation received Tyrosine Kinase Inhibitor (TKI) therapy; and the one patient who had received two cycles of ICIs prior to surgery continued with immunotherapy (Table 3).

After a median FUP of 8 (IQR:10) months, total body CT scans showed no pleural disease progression or MPE recurrence in any of the patients. However, five patients developed extrathoracic disease progression (Table 3).

## 4. Discussion

Only limited clinical data are available on the efficacy of intrathoracic aerosol chemotherapy using PITAC, primarily from a few case series [10,27,28,29,30,31]. The PITAC procedure was adapted from abdominal surgery, specifically from Pressurized Intra-Peritoneal Aerosol Chemotherapy (PIPAC). PIPAC was introduced for the management of unresectable PtM in end-stage patients, where it achieved encouraging outcomes in histological tumor regression and effective ascites control, while preserving quality of life (QoL) [11,32]. The procedure has demonstrated safety and feasibility, with minimal postoperative complications and low systemic toxicity [33].

Given the promising results of PIPAC and the lack of alternative treatments for patients with PC-NSCLC and MPE, we designed a pilot study to evaluate the feasibility and oncological potential of PITAC as a thoracic adaptation of PIPAC.

PITAC involves the nebulization of aerosolized chemotherapy within the thoracic cavity during standard thoracoscopy. The rationale is based on physical principles: aerosol particles are evenly distributed in a closed space, and the pressure gradient enhances drug penetration by overcoming tumor interstitial pressure. Compared to liquid solutions, aerosols offer a higher surface area-to-volume ratio, facilitating deeper tissue penetration at higher local drug concentrations [13]. Additionally, chemotherapeutics with sclerosing properties can induce uniform pleurodesis, potentially alleviating MPE-related symptoms. This minimally invasive approach allows for repeatability, low morbidity, and improved QoL [12]. PITAC, like PIPAC, has also proven safe in terms of occupational exposure, with no evidence of OR contamination by aerosolized drugs [33].

PITAC was first performed in 2012 by Reymond in Herne, Germany, in combination with PIPAC for patients with pleural and peritoneal metastases from various origins, including gastric, ovarian, and mesothelioma [30]. However, the patient group was highly heterogeneous.

Our study is the first to focus exclusively on PC-NSCLC patients with MPE, treated by specialized thoracic surgeons. The preliminary results are encouraging and align with findings from the few available series [27,30,31]. PITAC proved technically feasible and safe in our cohort. All patients tolerated the procedure well. No intraoperative complications occurred, no postoperative complications of CTCAE grade >3 were observed, and 30-day mortality was zero, findings consistent with earlier PITAC and PIPAC reports [29,30,31]. In contrast, Kuchen et al. described a more heterogeneous group, including advanced stages of different tumors. Only four patients received PITAC for isolated MPE, while six underwent combined PITAC and PIPAC for abdominal carcinomatosis. Their series reported one intraoperative bowel injury (during PIPAC), a 14.7% rate of postoperative complications, including two prolonged air leaks after PITAC, a longer hospital stay (8.5 ± 8.0 days vs. 5.4 ± 4.7 in our series), and a high 30-day mortality rate of 28.6% [29].

Regarding the chemotherapeutic regimen, previous studies used only cisplatin (10.5 mg/m^2^) and doxorubicin (2.1 mg/m^2^), based on a dose-escalation study for intraperitoneal chemotherapy in recurrent ovarian cancer with PtM [10,30,31,34]. We selected the same combination due to its established efficacy, favorable local pharmacokinetics, and frequent use in regional chemotherapy protocols. Cisplatin offers strong cytotoxic activity with low systemic absorption, while doxorubicin enhances tumor penetration and synergizes with cisplatin [35,36]. This combination has shown promising results in intrathoracic chemotherapy [8,37]. Carboplatin, by contrast, has demonstrated lower efficacy in aerosol applications [38]. We corroborate the administration of platinum-based drugs and anthracyclines because, in addition to direct cytotoxic and cytostatic effects, both cisplatin and doxorubicin possess sclerosing properties that contribute to pleural cavity obliteration by inducing fibrinous pleuritis, thereby reducing MPE recurrence [39]. As evidence of the facts, in our series, pleurodesis was achieved in all patients and remained effective for over six months. No pleural disease progression or MPE recurrence was observed on FUP imaging. However, all patients also received systemic treatments (chemotherapy, ICIs, or TKIs), making it difficult to isolate PITAC’s effect. Despite this, five of seven patients developed extrathoracic disease progression (three of whom were stage IVA—M1b at diagnosis) suggesting PITAC may have contributed significantly to locoregional disease control. These findings advocate for further evaluation of its potential systemic cytotoxic effects.

Conversely, the role of HITHOC in managing MPE remains limited [6,9]. HITHOC is usually employed alongside cytoreductive surgery (e.g., pleurectomy/decortication) or after resection of primary tumors like lung cancer, thymoma, and thymic carcinoma. These approaches are typically reserved for patients eligible for extensive surgical resections and are applied with curative intent in the context of resectable disease [8]. In contrast, the PITAC procedure leverages aerosolized drug delivery and CO_2_-induced intrapleural pressure to enhance drug distribution and tumor penetration. This method allows for lower doses of chemotherapeutic agents compared to HITHOC, thereby minimizing systemic absorption and reducing associated toxicities [9,29]. Therefore, PITAC represents a promising therapeutic option for patients who are unfit for radical surgery, offering a minimally invasive approach to achieve local tumor control with reduced systemic toxicity and improved tolerability.

To our knowledge, this is the first study focused on PITAC for PC-NSCLC, with or without MPE. To validate our findings, we also developed an in vitro model using primary cell cultures derived from patient tumor samples to evaluate PITAC’s antineoplastic activity.

Despite the small sample size, results from the 2D cultures obtained immediately post-PITAC were notable. No viable tumor cell growth was observed, demonstrating the cytotoxic and antiproliferative effect of pressurized aerosol chemotherapy.

No prior study has evaluated PITAC in vitro or in vivo for PC-NSCLC. Patient-derived primary cultures are a reliable preclinical model, closely mimicking the original tumor tissue [14,15]. Numerous studies have confirmed that in vitro chemosensitivity often correlates with clinical outcomes, suggesting these models may help personalize treatment in advanced-stage NSCLC. Personalized medicine approaches could benefit from treating patient-derived cultures with the same agents used in vivo, potentially guiding individualized therapy selection [24,25,26].

We also highlight the multidisciplinary approach of our study, which integrates clinical and radiological evaluation with in vitro testing of the chemotherapeutic regimen. Our preliminary results are promising and lay the groundwork for future studies using patient-derived tumor cells to optimize PITAC. A dose-escalation analysis of cisplatin and doxorubicin using an in vitro model that replicates PITAC will help define the most effective concentrations. A minimum enrollment of 30 patients is expected over the next 3 years in this ongoing phase 2 study. In the near future, this translational model could be adopted to test in vitro and/or in vivo chemosensitivity of patient’s tumor cells against a range of different potential therapeutic agents to select the best option and combination for each patient on a personalized basis.

Meanwhile, some limitations should be acknowledged; first, the small sample size and the absence of a control group should be considered when interpreting the study results. On the other hand, in the present work, we reported the preliminary results of a more extensive pilot study, which is still ongoing. In addition, PITAC is a new procedure, and our results should be independently confirmed. The requirement for environmental precautions may also be a limitation and further investigation is needed to justify wider use of PITAC, particularly compared to less complex methods. Neither survival probability nor predictive factors were evaluated because statistical significance could not be obtained according to the underpowered sample size. Lastly, the non-randomized design could limit the generalizability of the results because the selection bias might influence favorably for PITAC. Future randomized controlled trials are needed to validate our findings.

## 5. Conclusions

This pilot study demonstrated the feasibility, safety, and efficacy of PITAC in inducing palliative pleurodesis, with promising outcomes regarding local antineoplastic control. The absence of intraoperative complications, low morbidity, and effective pleurodesis at various time points underscore the potential of PITAC in revolutionizing loco-regional therapy for PC-NSCLC.

While the complexity of pleural metastases may limit PITAC’s efficacy, several aspects support its potential. PITAC ensures homogeneous aerosol distribution under pressure, enhancing drug penetration beyond superficial layers. In early pleural dissemination, lesions are often superficial, making PITAC particularly suitable. Preclinical data confirm aerosol infiltration into deeper tissue. PITAC also complements systemic therapies, offering synergistic local control. It should thus be considered part of a broader multimodal treatment strategy.

An in vivo and in vitro validation of this technique could pave the way for a new approach to managing these particularly frail metastatic patients, as the ability to locally administer chemotherapy may change the natural history of the disease without causing systemic side effects. Furthermore, due to its relatively low cost, PITAC may represent a highly sustainable procedure in terms of both clinical outcomes and socio-economic impact on the healthcare system.

As the first report focused exclusively on PC-NSCLC, this work lays the groundwork for future investigations, encouraging the pursuit of personalized medicine approaches and advancing the field of loco-regional therapy for thoracic malignancies with larger, controlled trials to validate the observed outcomes.

## Figures and Tables

**Table 1:** Patients’ pre-operative characteristics.

Characteristic	Patients (n; %)
** Gender**	
Male	4 (57%)
Female	3 (43%)
**Median Age** (years, IQR)	65 (19)
** ECOG score**	
1	3 (43%)
2	4 (57%)
** Comorbidities**	
COPD	3 (43%)
Hypertensive heart disease	5 (72%)
Heavy smoking	3 (43%)
Cerebrovascular disease	2 (28%)
** Stage ^a^**	
IVA—M1a	4 (57%)
IVA—M1b (bone)	3 (43%)

Note: n, number; IQR, interquartile range; ECOG, Eastern Oncology Cooperative Group; COPD, chronic obstructive pulmonary disease; ^a^ according to the eight edition of TNM classification for lung cancer.

**Table 2:** Major immunohistochemical and molecular characteristics of lung adenocarcinomas.

Characteristic	Patients (n; %)
TTF-1 (+) TTF-1 (−)	6 (86%) 1 (14%)
CK-7 (+) CK-7 (−)	4 (57%) 3 (43%)
p40 (+) p40 (−)	1 (14%) 6 (86%)
Calretinin (+) Calretinin (−)	2 (28%) 5 (72%)
CK-Pan (+) CK-Pan (−)	4 (57%) 3 (43%)
Napsin A (+) Napsin A (−)	5 (72%) 2(28%)
BerEP4 (+) BerEP4 (−)	5 (72%) 2 (28%)
EGFR exon 19 deletion	2 (28%)
KRAS G12D mutation	2 (28%)
KRAS G12F mutation	1 (14%)

Note: n, number; (+), positivity; (−) negativity; TTF-1, Thyroid Transcription Factor 1; CK, cytokeratin; EGFR, Epidermal Growth Factor Receptor; KRAS, Kirsten Rat Sarcoma Virus.

**Table 3:** Treatment and disease progression.

Characteristic	Patients (n; %)
** Site of disease progression**	
None	2 (29%)
Bone	3 (43%)
Brain	1 (14%)
Liver	1 (14%)
** Post-operative systemic therapy**	
Platinum-based CT + ICI (Pembrolizumab)	3 (43%)
Platinum-based CT + ICI (Pembrolizumab) + RT (bone)	1 (14%)
TKI (Osimertinib)	2 (29%)
ICI (Atezolizumab) + second-line platinum-based CT *	1 (14%)

Note: n, number; CT, chemotherapy; ICI, immune checkpoint inhibitor; TKI, tyrosine kinase inhibitor. * The only patient who underwent preoperative systemic therapy (with ICI, atezolizumab).

## Data Availability

The article’s underlying data will be shared on reasonable request to the corresponding author.

## Supplementary Materials

The following supporting information can be downloaded at: https://www.mdpi.com/article/10.3390/mps8030051/s1, Supplemental File S1: The CONSORT checklist.
